# The Na^+^–H^+ ^exchanger-1 induces cytoskeletal changes involving reciprocal RhoA and Rac1 signaling, resulting in motility and invasion in MDA-MB-435 cells

**DOI:** 10.1186/bcr922

**Published:** 2004-09-13

**Authors:** Angelo Paradiso, Rosa Angela Cardone, Antonia Bellizzi, Anna Bagorda, Lorenzo Guerra, Massimo Tommasino, Valeria Casavola, Stephan J Reshkin

**Affiliations:** 1 Laboratory of Clinical & Experimental Oncology, Oncology Institute of Bari, Bari, Italy; 2 Department of General and Environmental Physiology, University of Bari, Bari, Italy; 3 International Agency for Research on Cancer, World Health Organization, Unit of Infection and Cancer, Lyon, France

**Keywords:** cytoskeleton, metastasis, NHE1, invasion, Rho GTPases

## Abstract

**Introduction:**

An increasing body of evidence shows that the tumour microenvironment is essential in driving neoplastic progression. The low serum component of this microenvironment stimulates motility/invasion in human breast cancer cells via activation of the Na^+^–H^+ ^exchanger (NHE) isoform 1, but the signal transduction systems that underlie this process are still poorly understood. We undertook the present study to elucidate the role and pattern of regulation by the Rho GTPases of this serum deprivation-dependent activation of both NHE1 and subsequent invasive characteristics, such as pseudopodia and invadiopodia protrusion, directed cell motility and penetration of normal tissues.

**Methods:**

The present study was performed in a well characterized human mammary epithelial cell line representing late stage metastatic progression, MDA-MB-435. The activity of RhoA and Rac1 was modified using their dominant negative and constitutively active mutants and the activity of NHE1, cell motility/invasion, F-actin content and cell shape were measured.

**Results:**

We show for the first time that serum deprivation induces NHE1-dependent morphological and cytoskeletal changes in metastatic cells via a reciprocal interaction of RhoA and Rac1, resulting in increased chemotaxis and invasion. Deprivation changed cell shape by reducing the amount of F-actin and inducing the formation of leading edge pseudopodia. Serum deprivation inhibited RhoA activity and stimulated Rac1 activity. Rac1 and RhoA were antagonistic regulators of both basal and stimulated tumour cell NHE1 activity. The regulation of NHE1 activity by RhoA and Rac1 in both conditions was mediated by an alteration in intracellular proton affinity of the exchanger. Interestingly, the role of each of these G-proteins was reversed during serum deprivation; basal NHE1 activity was regulated positively by RhoA and negatively by Rac1, whereas RhoA negatively and Rac1 positively directed the stimulation of NHE1 during serum deprivation. Importantly, the same pattern of RhoA and Rac1 regulation found for NHE1 activity was observed in both basal and serum deprivation dependent increases in motility, invasion and actin cytoskeletal organization.

**Conclusion:**

Our findings suggest that the reported antagonistic roles of RhoA and Rac1 in cell motility/invasion and cytoskeletal organization may be due, in part, to their concerted action on NHE1 activity as a convergence point.

## Introduction

Tumour invasion and metastasis associated with neoplastic progression are the major causes of cancer deaths. The invasive process occurs through a complex series of interactions with the host tissue, resulting in infiltration and penetration of normal tissue by cancer cells [1]. Recent advances have highlighted the importance of the acid component of the tumour microenvironment in driving invasive capacity and subsequent malignant progression [2-4]. The activity of the Na^+^–H^+ ^exchanger (NHE) isoform 1 is known to play a role in acidifying the tumour microenvironment [5], and the nutrient-deprived conditions that are common to the tumour microenvironment activate tumour cell NHE1, which in turn stimulates increased motility and invasive capability [6]. This role played by Na^+^–H^+ ^exchanger activity in driving tumour cell motility has been corroborated in transformed renal cells [7,8] and in ascites hepatoma cells [9], emphasizing the importance of understanding the mechanisms that control Na^+^–H^+ ^exchanger activity in tumour cells.

The Rho family of small GTPases has been shown to play a major role in regulating the rearrangement of the actin cytoskeleton in response to cell stimulation [10] and to be involved in the regulation of a variety of other cellular processes, such as organization of the microfilamental network, cell–cell contact, motility and apoptosis [11]. Early studies in fibroblasts suggested that Cdc42, Rac1 and RhoA are organized in hierarchical cascades in which activated Cdc42 activates Rac1, in turn activating RhoA [10]. It is now known that these G-proteins can be organized in many other ways. For example, Rac1 or Cdc42 have been shown to oppose or modify RhoA action in the regulation of a number of processes, including actin cytoskeleton remodelling [12], axon guidance [13], dendrite branching [14] and cell migration [15-17]. Thus, it is clear that the interactions and associations of the various members of the Rho family are cell/tissue-specific or function-specific, or both. Despite the evidence that Rho GTPases are overexpressed in tumours [18] and play an important role in mitogenesis, proliferation and invasiveness [19], our understanding of how they interact among themselves in tumour cells to regulate these processes is still incomplete and represents an important issue in oncology [20].

Molecular control of actin organization is probably at the core of cell motility, and it is of importance that we gain an understanding of the mechanisms that underlie the regulation of actin dynamics so that we may appreciate how motility is regulated. Recent studies conducted in smooth muscle cells have led to a model (for review see [21,22]) that associates RhoA with contraction via activation of Rho kinase (ROCK) and ROCK-dependent phosphorylation of myosin phosphatase, thereby inactivating it and resulting in an increase in the phosphorylation state of myosin light chain and enhancement of myosin binding to actin filaments. Rac1 via activation of the p21-activated kinase (PAK) antagonizes this process by blocking the activity of myosin light chain kinase. As recently discussed [23], this mechanism pertains to smooth muscle cell contraction and cannot fully explain the effects of Rho family proteins on the actin cytoskeleton in other cell types, such as epithelial cells. Two other mechanisms have been described in which Rac1 downregulates RhoA activity via a redox-dependent mechanism [17] and by stimulating RhoA degradation via Smurf1 [24]. Recently, however, a further possible cross-regulatory mechanism has emerged. Pioneering studies in fibroblasts have shown that NHE1, a regulator of intracellular pH (pHi), can play a direct role in controlling actin dynamics and subsequent motility through a protein–protein interaction with the cytoskeletal adaptor protein ezrin, and that, in those cells, RhoA-dependent modulation of cytoskeletal dynamics and motility occurred via direct regulation of NHE1 activity [25,26]. There is increasing evidence that activity of the NHE1 is essential for motility in various cell types [6-9,26]. Accordingly, the questions are whether RhoA and Rac1 reciprocally regulate motility in tumour cells of epithelial origin, and if so then do they act via a coordinated regulation of NHE1 activity?

We previously reported that serum deprivation, a common component of the tumour microenvironmental, stimulates NHE1 in human epithelial breast cancer cells and drives increased cellular motility and invasive ability via the activated NHE1 [6]. In light of this essential role played by NHE1 in regulating motility in these cells, the present study was undertaken to characterize the role and pattern of regulation by Rho GTPases of this serum deprivation-dependent activation of both NHE1 and subsequent basic invasive characteristics, such as pseudopodia and invadiopodia protrusion, directed cell motility and penetration of normal tissues [27,28]. The study was conducted in a well characterized human mammary epithelial cell line that represents a late phase in metastatic progression, namely MDA-MB-435 [6]. We observed that serum deprivation inhibits RhoA activity and stimulates Rac1 activity and, using dominant negative and constitutively active mutants, that Rac1 and RhoA are antagonistic regulators of tumour cell NHE1 activity. As was observed in the regulation of this phenomenon by phosphoinositide-3 kinase (PI3K) [6], a reversal of RhoA and Rac1 regulatory action on NHE1 activity found in serum replete conditions was found during serum deprivation-dependent upregulation of NHE1. Although serum deprivation reversed the regulatory actions of Rac1 and RhoA on NHE1 activity, the basic pattern of antagonism of action between these two G-proteins was maintained. Furthermore, the same pattern of RhoA and Rac1 regulation found for NHE1 activity was observed in both basal and serum deprivation-dependent increases in cell invasion and motility. Together with the recent reports demonstrating the direct role of NHE1 in controlling actin dynamics in fibroblasts [25,26], our findings suggest that the reported antagonistic roles of RhoA and Rac1 in cell motility and cytoskeletal organization may also be due, in part, to their concerted action on NHE1 activity as a convergence point.

## Methods

### Cells and construction of expression vectors containing RhoA mutants

MDA-MB-435 cells were cultured as previously described [6]. Minus serum growth medium was complete Dulbeccos modified Eagle's medium (DMEM; GibcoBRL, Milano, Italy) without serum, in which the osmolarity, if necessary, had been adjusted to be identical to the plus serum medium by the addition of the necessary amount of mannitol. To deprive cells of serum, monolayers that had grown to confluency in complete DMEM with 10% foetal calf serum were washed two times in the minus serum DMEM and then the same volume of minus serum medium was added and the monolayers replaced in the incubator. After the indicated times the experiments were conducted.

**Figure 1:**
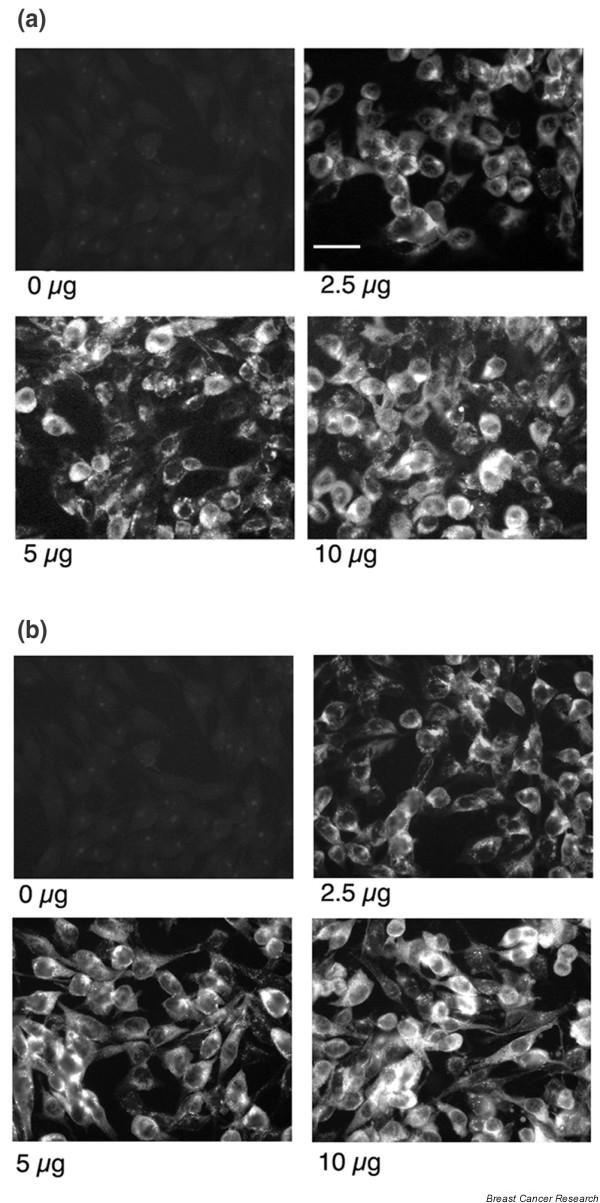
Efficiency of transfection with Rho family constructs. MDA-MB-435 cell monolayers were transfected with variable concentrations of the pBabe vector containing either the dominant negative **(a) **N17Rac1 or **(b) **17NCdc42 construct. Forty-eight hours later the monolayers were fixed with 4% paraformaldehyde, and the expression of the haemagglutinin (HA) tag visualized by immunofluorescent microscopy with an anti-HA antibody. The percentage of cells expressing HA-tagged N17Rac1 increased in a concentration-dependent manner and reached approximately 90% when the cells were transfected with 10 μg plasmid cDNA.

pCEFL plasmids containing the dominant negative N19RhoA, N17Rac1 and N17Cdc42 mutants were kindly provided by Dr PP Di Fiore (European Institute of Oncology, Milan, Italy) and pEXV plasmids containing the constitutively active V14RhoA and V12Rac1 mutants by Dr MH Symons (Picower Institute for Molecular Research, Manhasset, NY, USA). These cDNAs were subcloned into the pBabe puro expression vector containing a haemagglutinin (HA) tag. Ten micrograms of plasmid cDNA or empty vector was incubated with 100 μl LipoTaxi reagent (Stratagene, La Jolla, CA, USA) in 1 ml of simple DMEM growth medium for 30 min at room temperature. Serum was added to 3% and 200 μl of this mixture was pipetted onto confluent monolayers on glass coverslips and placed in an incubator at 5% CO_2 _and 37°C for 6 hours. This mixture was then replaced with fresh complete medium (10% serum) for 24 hours in normal growth conditions. Cells were then treated and NHE1 activity, motility, or invasion were measured. The level of transfection was determined using Western blot of whole cell extracts using anti-HA antibody (Santa Cruz, Santa Cruz, CA, USA). The percentage of transfection with different concentrations of cDNA (0, 2.5, 5 and 10 μg plasmid cDNA) was evaluated by immunofluorescence microscopy of cell monolayers on coverslips using the same anti-HA antibody. As shown in Fig. 1 the transfection efficiency for both the dominant negative (dn) N17Rac1 cDNA and N19RhoA cDNA was commensurate with cDNA concentration and reached approximately 90% when cells were transfected with 10 μg plasmid cDNA. The transfection efficiencies observed with the other cDNAs utilized in the study were very similar to those for N19RhoA and N17Rac1 (data not shown).

### Intracellular pH and H^+ ^efflux rate determinations

Intracellular, cytoplasmic pH (pHi) was measured spectrofluorimetrically at 37°C with the fluorescent pH sensitive probe 2',7'-bis(carboxyethyl)-5, 6-carboxy-fluorescein (BCECF), and trapped intracellularly in cell monolayers grown on glass coverslips as previously described [6]. NHE1 activity was measured by monitoring pHi recovery after an intracellular acid load produced with the NH_4_Cl prepulse technique. The initial rate of Na^+^-dependent alkalinization was determined by linear regression analysis of the first 15 points taken at 4 s intervals after the readdition of sodium. The use of CO_2_/HCO_3 _free solutions minimizes the likelihood that Na^+^-dependent HCO_3 _transport was responsible for the observed changes in pHi. The pHi dependence of intracellular buffer capacity (βi; i.e. the buffering power of all non-HCO_3_, non-CO_2 _buffers) was computed using the NH_4 _pulse method, and the actual activity of the exchanger in terms of proton flux rate (mmol/l H^+^/min) was determined by multiplying the rates of pHi change by the cells' intrinsic buffering capacity (βi) at the pHi at which the measurement was taken. Preliminary experiments demonstrated that 2 μmol/l of the specific NHE1 inhibitor 5-(N, N-dimethyl)-amiloride (DMA; Sigma, Milano, Italy) almost completely blocked NHE1 activity, whereas transfection with empty pBabe vector had no effect on NHE1 activity (data not shown).

### Motility and invasion

A quantitative measure of the degree of *in vitro *motility of the cells was obtained in BW25 Boyden Chambers (Neuro Probe Inc., Cabin John, MD, USA) using 8 μm polycarbonate membranes (Poretics, Livermore, CA, USA) coated with 5 μg collagen I as previously described [6]. After 4 hours of incubation, cells were fixed and stained (DiffQuick; Baxter, Oakland, CA, USA), those cells that had not transversed the filter were removed, and randomly chosen fields were photographed at 100× magnification using a Nikon Eclipse E800 microscope equipped with an MRC-1024 imaging system (Bio-Rad Laboratories, Milano, Italy). The number of cells traversing the filter in four random fields for each filter were counted from these images.

A quantitative measure of the degree of *in vitro *invasion was measured as the ability to infiltrate into live, confluent MCF-10A monolayers, essentially as previously described [6,29]. MDA-MB-435 cells were metabolically loaded with ^3^H-thymidine for the 24 hours prior to the experiment, cells were trypsinized, and a standard curve of incorporated ^3^H-dT/cell measured for each treatment. Tumour cells (80,000) were added in suspension to the complete medium of the confluent MCF-10A monolayers. Culture dishes were returned to the incubator for 8 hours and then unattached cells were removed by vigorous washing and mechanical agitation two times with phosphate-buffered saline (PBS). The number of MDA-MB-435 cells that had invaded the MCF-10A monolayer was calculated by measuring the incorporated ^3^H-thymidine for each MCF-10A monolayer in a Packard TopCount NXT^® ^microplate scintillation counter (Packard Instruments, Inc., Palo Alto, CA, USA), and the number of cells present computed using the standard curve of disintegrations per min/cell for each treatment. Experiments standardizing this assay against the Boyden Chamber matrigel assay demonstrated high correlation between the two techniques.

### Expression and activity of RhoA and Rac1

RhoA and Rac1 activity were assessed using the RhoA-binding domain of Rhotekin or Rac1-binding domain of PAK-1, respectively, in kits supplied from Upstate Biotechnology (Lake Placid, NY, USA). In brief, 3 × 10^6 ^cells were plated onto 10 cm cell culture dishes and after 24 hours they were treated as indicated. After the indicated time, cells were extracted using RIPA buffer (50 mmol/l Tris, pH 7.2, 500 mmol/l NaCl, 1% Triton X-100, 0.5% sodium deoxycholate, 1% SDS, 10 mmol/l MgCl_2_, 0.5 μg/ml leupeptin, 0.7 μg/ml pepstatin, 4 μg/ml aprotinin, and 2 mmol/l PMSF). After centrifugation at 14,000 *g *for 3 min, the extracts were incubated for 45 min at 4°C with glutathione beads coupled with glutathione S-transferase (GST)–RBD (Rho-binding domain of Rhotekin) fusion protein or GST-PAK-1 (Upstate Biotechnology), and then washed three times with Tris buffer (pH 7.2), containing 1% Triton X-100, 150 mmol/l NaCl and 10 mmol/l MgCl_2_. The RhoA or Rac1 content in these samples or in 50 μg protein of cell homogenate was determined by immunoblotting samples using rabbit anti-RhoA antibody (Santa Cruz) or anti-Rac1 antibody (Upstate Biotechnology).

### Fluorescence microscopy

For analysis of actin cytoskeleton organization, cells were plated on coverslips until they were approximately 60% confluent, at which time they were transfected with empty vector or dn-RhoA or dn-Rac1 and, after 24 hours of incubation, they were either subjected or not subjected to serum starvation in the presence or absence of 2 μmol/l DMA. After 24 hours, cells were fixed with 4% paraformaldehyde in PBS for 15 min, and after residual formaldehyde had been quenched with 50 mmol/l NH_4_Cl in PBS for 10 min the cells were permeabilized with 0.2% Triton X-100 in PBS for 10 min. Filamentous actin (F-actin) was stained with fluorescein isothiocyanate-conjugated phalloidin (Sigma) in PBS (0.5 U/ml) for 1 hour, whereas HA-positive cells were determined with anti-HA antibody (Santa Cruz) at a 1:100 dilution. Microscopy was performed using an MRC-1024 imaging system (Bio-Rad Laboratories) equipped with a Nikon Eclipse E800 microscope and a Nikon Plan Apo 40-by-1.0 or 60-by-1.4 oil immersion objective lens.

### Analysis of F-actin content

Actin polymerization was detected using fluorescent phalloidin and analyzed by confocal microscopy and fluorescence activated cell sorting analysis. Analytic flow cytometric measurements were performed basically as previously described [30], using a Hewlett-Packard 9153C flow cytometer with argon laser excitation at 488 nm and detection through a 515–540 nm bandpass filter. Ten thousand cells in each sample were analyzed. For determination of F-actin amount the gate was defined as corresponding to scatter parameters on a dot plot of viable control cells (nondeprived). Phalloidin–fluorescein isothiocyanate fluorescence intensities from the gated population were presented as a histogram and the geometrical mean value was used as a measure of F-actin content.

### Kinetic and statistical analysis

The kinetic coefficients for proton dependency of exchange activity were estimated using nonlinear curve-fitting regression utilizing the iterative Marquardt procedure on the KALIDIOGRAPH^® ^(Abelbeck Software, Reading, PA, USA) program. The proton data were fitted to the Hill equation – V = (Vmax [H]^n^)/(Km + [H]^n^) – in which V is the experimental transport velocity, Vmax is the calculated maximum transport velocity, [H] is the cytosolic proton concentration, Km is the apparent affinity for protons, and n is the apparent Hill coefficient.

## Results

### NHE1 lies upstream of serum deprivation-dependent actin cytoskeletal reorganization

**Figure 2:**
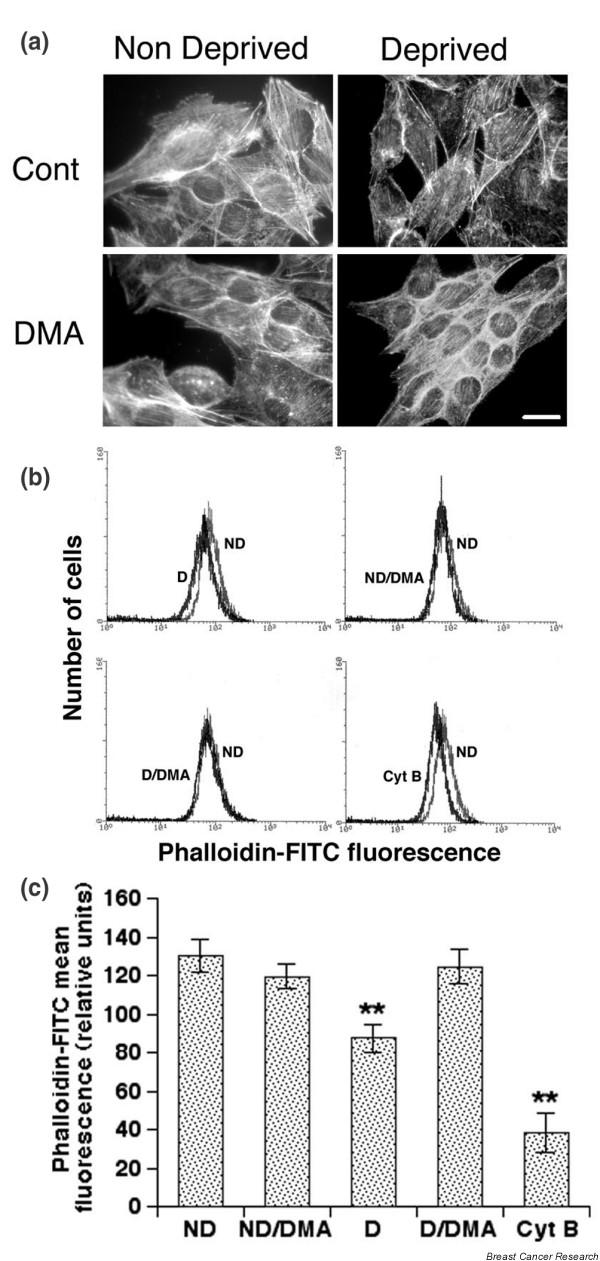
Role of Na^+^–H^+ ^exchanger (NHE)1 in serum deprivation-dependent rearrangement of the actin cytoskeleton. **(a) **To examine the role of NHE1 in serum deprivation-induced reorganization of the actin cytoskeleton underlying motility and invasive ability, MDA-MB-435 cell monolayers were left in fresh whole growth medium (nondeprived [ND]) or were serum deprived (D) in the presence or absence of 2 μmol/l of the specific NHE1 inhibitor 5-(N, N-dimethyl)-amiloride (DMA) for 1 day. The fluorescent photomicrographs show cell groups at a magnification of 40× (bar = 10 μm). **(b) **typical one-parameter fluorescence distributions of MDA-MB-435 cells labelled with phalloidin–fluorescein isothiocyanate in the above experimental conditions. The lighter coloured curve is from control, ND cells. Cyt B is the fluorescence distribution after treatment with the actin cytoskeleton disruptor cytochalasin B (5 μmol/l for 15 min). **(c) **Relative intracellular F-actin content measured by flow cytometry in MDA-MB-435 cells in the above experimental conditions. Mean ± standard error. *n *= 9; **P < 0.001 versus ND cells.

We previously showed that serum deprivation selectively stimulates NHE1 activity in breast cancer cells and their subsequent NHE1-dependent motility and invasion [6]. Recently, mounting evidence has shown that NHE1-dependent regulation of motility in normal cells occurs by direct NHE1-dependent reorganization of the actin cytoskeleton [25,26]. To evaluate whether NHE1 is upstream of actin reorganization during serum deprivation in cancer cells, we followed the serum deprivation-dependent remodelling of the F-actin cytoskeleton (Fig. 2a) and quantity of F-actin (Fig. 2b,2c) in the presence or absence of the specific NHE1 inhibitor DMA. As can be seen in Fig. 2a, control cells were primarily fusiform in shape with thin, uniform, parallel actin filaments (stress fibres) departing from single points and extending throughout the length of the cell. Serum deprivation provoked a complex and dramatic reorganization of the actin cytoskeleton, resulting in a reduction in the number of stress fibres with an uneven thickening of the remaining stress fibres. Consistent with the model proposed by the group of Barber [25,26], inhibition of NHE1 during serum deprivation with 2 μmol/l DMA completely abrogated the observed serum deprivation-dependent cytoskeletal reorganization, and depolymerization suggesting that stimulation of NHE1 is indeed up-stream of actin cytoskeletal reorganization. As can be seen in Fig. 2c, treatment with the actin cytoskeleton disruptor cytochalasin B (5 μmol/l for 15 min) reduced the amount of F-actin by approximately 75%. The lack of significant inhibitory effect by cytochalasin B treatment on NHE1 activity (absence versus presence of cytochalsin B: 16.2 ± 2.7 versus 20.6 ± 4.7 mmol/l intracellular H^+ ^concentration/min [27% increase], *n *= 7; *P *= 0.58) further supports this hypothesis [31].

### Serum deprivation activates Rac1 and inactivates RhoA

The usual model for RhoA-dependent alterations in motility is linked to RhoA action upon the actin cytoskeleton via the phosphorylation/inactivation of myosin phosphatase by the RhoA effector p160ROCK [21,22]. However, mounting evidence has suggested the existence of an alterative system in which RhoA/ROCK directly alters NHE1 activity [32] followed by the NHE1-dependent reorganization of the actin cytoskeleton [25,26]. It was recently reported that the tight junction protein NZO-3 increases kidney cell motility via a decrease in stress fibre number due to an inhibition of RhoA activity [33], and dihydromotuporamine C decreases cancer cell motility and invasion via an increase in stress fibre number that was due to a stimulation of Rho activity [34].

**Figure 3:**
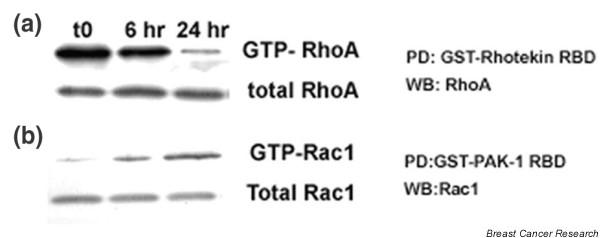
Serum deprivation activates Rac1 and inactivates RhoA without changes in total cellular expression. MDA-MB-435 cells were nondeprived (t0) or deprived of serum for 6 or 24 hours and cell extracts were assayed for **(a) **Rhotekin or **(b) **p21-activated kinase (PAK-1)-binding activity, respectively, as described in the Methods section. Representative immunoblots from these experiments are shown. For these experiments, the RhoA bound to the RBD or Rac1 bound to PAK-1 (upper panels) were normalized to the total RhoA or Rac1 content of cell extracts (lower panels). GST, glutathione-S-transferase; RBD, Rho-binding domain of Rhotekin (upper panel) or Rac-binding domain (lower panel); WB, Western blot.

As a starting point for our study of the regulatory pattern of RhoA and Rac1 in the serum deprivation regulation of NHE activity and the migration/invasion of MDA-MB-435 cells, we measured the effect of serum deprivation on the activation status of RhoA and Rac1. To assess RhoA and Rac1 activation states, we used the GST fusion proteins of RBD to capture GTP-bound RhoA, and PAK-1 Rac-binding domain to capture GTP-bound Rac-1 from cell extracts. As shown in Fig. 3, serum deprivation resulted in a significant decrease in the amount of RhoA retained by RBD (panel a) and in a significant increase in Rac1 retained by PAK-1 Rac-binding domain (panel b). In contrast, serum deprivation had no effect on total cellular expression of either RhoA or Rac-1, demonstrating that it is indeed changes in their activity and not expression that drives their regulatory control of NHE activity. A similar pattern of inverse correlation between levels of active RhoA and active Rac1 was previously described in epithelial cells and fibroblasts [35,36].

### RhoA and Rac1 reciprocally regulate the activity of NHE1 in basal and serum deprivation stimulated conditions

**Figure 4:**
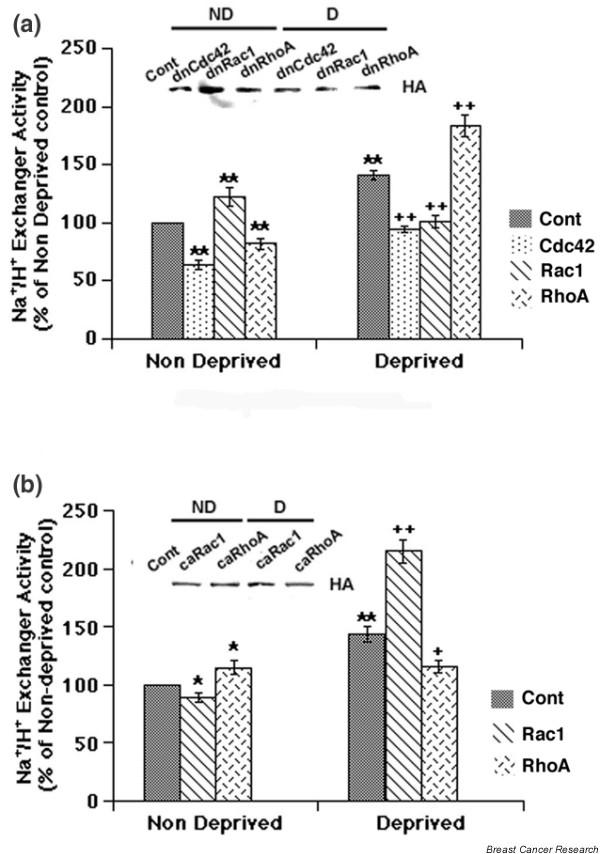
Effect of Rho family mutants on the basal and serum deprivation stimulation of Na^+^–H^+ ^exchanger (NHE)1 activity. To determine the role of Rho family G-proteins in the regulation of basal and serum deprivation stimulated Na^+^–H^+ ^exchanger (NHE)1 activity, monolayers were transfected with empty pBabe vector or vector containing mutated Rho family genes. After 24 hours, medium was replaced with either serum replete or deplete medium for an additional 24 hours. Intracellular pH recoveries were measured and the initial rate of the NHE1 activity calculated as Na^+^-dependent H^+ ^efflux, as described in the Methods section. All measurements were conducted in nominally HCO_3_-free, HEPES-buffered solutions in order to measure only the NHE. **(a) **Effect of dominant negative (dn) Rho family mutants on the serum deprivation-dependent upregulation of NHE1 activity. **(b) **Effect of constitutively active (ca) RhoA and Rac1 mutants on the serum deprivation-dependent upregulation of NHE1 activity. Data for control cells are shown in the gray solid bars, whereas the stippled bars represent data for Cdc42, stripped bars represent data for Rac1 and hatched bars represent data for RhoA. Data are expressed as means ± standard error for between 9 and 20 observations in each condition. *P < 0.01, **P < 0.001 versus control values; ^+^*P *< 0.01, ^++^*P *< 0.001 versus deprived values. Insets show the level of transfection of haemagglutinin (HA)-tagged cDNA in a typical experiment by Western blot of whole cell extracts using anti-HA antibody.

To address the question of whether Rho proteins are involved in the regulation of tumour cell NHE1 activity, we compared the effect of transient transfection of HA-tagged dn (Fig. 4a) or constitutively active (ca; Fig. 4b) mutants of Rho family proteins on both the basal activity of the NHE1 and its activation by serum deprivation. The relative level of transfection was determined using Western blot of whole cell extracts using anti-HA antibody (the inserts in the respective bar graphs in Fig. 4). The transfection efficiency for the various cDNAs was commensurate with cDNA concentration and reached approximately 90% when the cells were transfected with 10 μg plasmid cDNA (see the Methods section, above).

In cells not subjected to serum deprivation, the basal level of NHE1 activity was regulated reciprocally by these mutant constructs; specifically, NHE1 was slightly but significantly stimulated by inactivation of Rac1 induced by transfection with dn-N17Rac1 (stripped bar), and slightly but significantly inhibited by inactivation of RhoA induced by transfection with dn-N19RhoA (hatched bar). In serum deprived conditions NHE1 activity was still regulated reciprocally but the role of these G-proteins was reversed; inactivation of RhoA with N19RhoA potentiated the serum deprivation-dependent stimulation of the NHE1 (hatched bar), whereas inactivation of Rac1 with N17Rac1 blocked this stimulation (stripped bar). Inactivation of Cdc42 with dn-N17Cdc42 inhibited NHE1 activity to a similar degree in both nondeprived and deprived conditions (stippled bars).

These data suggest that RhoA and Rac1 play antagonistic roles in the regulation of both basal and serum deprivation-induced NHE1 activity, and that there is a reversal of their regulatory action with serum deprivation. To confirm this hypothesis, cells were transfected with constitutively active mutants of RhoA and Rac1. Activating RhoA with V14RhoA blocked serum deprivation-mediated stimulation of the NHE1 whereas the constitutively active V12Rac1 potentiated NHE1 stimulation by serum deprivation (Fig. 4b). Treating the cells with either a RhoA-specific pharmacological inhibitor (C3 exotoxin) or activator (CNF-1) produced a pattern identical to that observed with the RhoA mutated constructs (data not shown).

**Figure 5:**
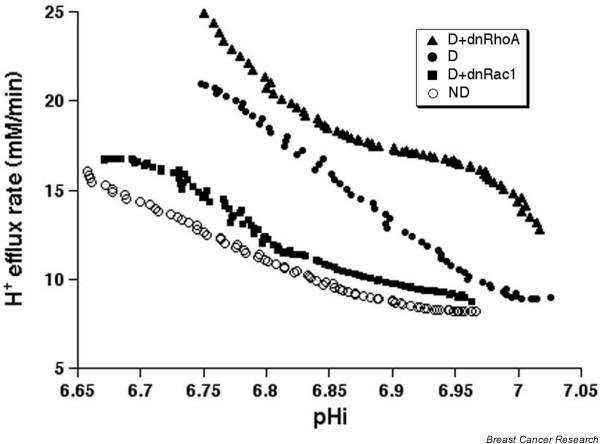
Effect of dominant negative (dn) RhoA and Rac1 mutants on the intracellular (pHi) dependence of net acid extrusion rate. To examine the mechanism of RhoA and Rac1 modification of the serum deprivation-dependent increase in Na^+^–H^+ ^exchanger (NHE)1 activity, the intracellular pH (pHi) dependence of NHE1 activity (Δmmol/l H^+ ^_i_/minute) in MDA-MB-435 cell monolayers was measured. Monolayers were left in fresh whole growth medium (open circles) or serum deprived (closed circles) plus dn-N19RhoA (triangles) or dn-N17Rac1 (squares) for 24 hours. Results from a typical experiment are shown. The plots were computed as described in the Methods section. H^+ ^_i_, intracellular proton concentration.

NHE1 activity is finely regulated by intracellular proton concentration via an allosteric proton regulatory site, and we previously showed that serum deprivation upregulated tumour cell NHE activity via an increased H^+ ^affinity of this site, resulting in an alkaline shift of the pK value for intracellular H^+ ^[6]. To determine whether RhoA and Rac1 modulate the serum deprivation-dependent upregulation of the NHE1 via this same mechanism, we analyzed the effect of the dominant negative mutants of RhoA or Rac1 on the dependence of NHE1 activity on pHi in serum deprived cells, as previously described [6]. Fig. 5 shows the relationship of NHE1 activity (Δmmol/l intracellular H^+ ^concentration/min) to pHi in 24 hr serum complete (open circles) and serum deprived cells minus (closed circles) or plus transfection with dn-N17Rac1 (squares) or dn-N19RhoA (triangles) in a typical experiment. Serum deprivation shifted the pK value for intracellular H^+ ^to alkaline values (alkaline shift) and increased the slope of the relationship. The observed alkaline shift in serum deprived conditions in the tumour cells is consistent with an increased capacity for net acid extrusion. In the cells transfected with dn-N17Rac1 (squares) serum deprivation no longer had any significant effect on the alkaline shift produced by serum deprivation, whereas transfection with dn-N19RhoA potentiated this alkaline shift. Kinetic analysis on five independent experiments for each treatment demonstrated that the Vmax and H^+ ^affinity increased in MDA-MB-435 cells upon serum deprivation (Table 1) without a change in the Hill coefficient (n_app _remained approximately 2). Also, transfection with dn-N17Rac1 blocked the serum deprivation-dependent kinetic shifts whereas transfection with dn-N19RhoA potentiated them. These data suggest that one of the mechanisms by which RhoA and Rac1 regulate the serum deprivation-dependent increases in NHE1 activity is by modifying the affinity of the internal proton regulatory site of the NHE1 for protons.

### Effect of dominant negative RhoA and Rac1 on serum deprivation-induced alterations in cell shape and actin organization

**Figure 6:**
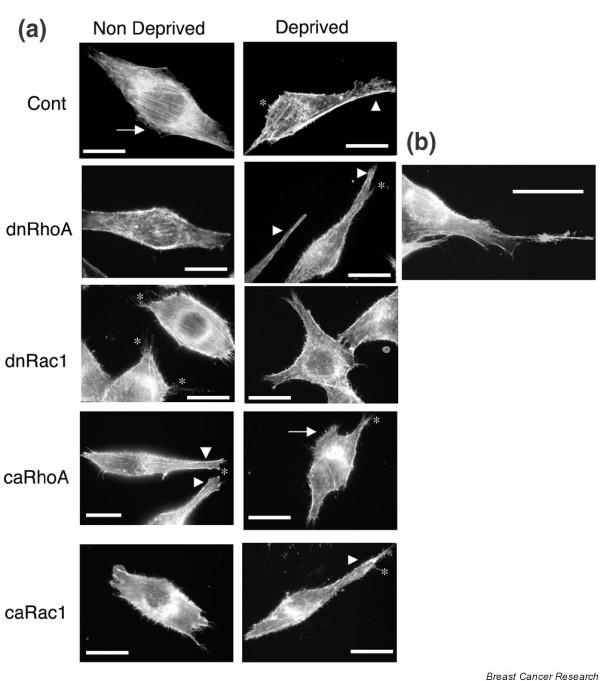
Role of RhoA and Rac1 in serum deprivation-dependent rearrangement of the actin cytoskeleton and cell shape. To examine the role of RhoA and Rac1 in serum deprivation-induced reorganization of the actin cytoskeleton underlying motility and invasive ability, MDA-MB-435 cell monolayers were **(a) **left in fresh whole growth medium (nondeprived) or were serum deprived (deprived) for 1 day after transfection with empty pBabe vector (Cont) or with dominant negative (dn)-N19RhoA, constitutively active (ca)-V14RhoA, dn-Rac1 or V12Rac1 inserted in the same vector (bar = 10 μm). Arrows indicate the presence of lamellipodia whereas pseudopodia are indicated by arrowheads and invadopodia along the cell surface or at the end of an dominant leading edge pseudopodia are indicated by an asterist. **(b) **The complex actin organization of a long, dominant leading edge pseudopodia extension in a cell transfected with dn-RhoA and deprived of serum at a higher magnification (bar = 5 μm).

In order to determine the alterations in F-actin cytoskeletal organization associated with the above changes in cell shape, we next incubated the cells with tetramethylrhodamine 5-isothiocyanate (TRITC)-labelled phalloidin in order to stain F-actin in the different treatments. As can be seen in Fig. 6a, control cells were primarily fusiform in shape with thin, uniform, parallel actin filaments (stress fibres) departing from single points and extending throughout the length of the cell, with the presence of lamellipodia (arrow), but there were few dominant, elongated pseudopodia and few actin containing, fine cell extensions (termed invadopodia [37,38]). Serum deprivation provoked a complex and dramatic reorganization of the actin cytoskeleton that resulted in a reduction in number of stress fibres and lamellipodia, with an uneven thickening of the remaining stress fibres and an increase in both dominant leading-edge pseudopodia (arrowhead) observed at the ends of the cells and in invadopodia (asterisk). Interestingly, transfection with mutant constructs that increase either basal or deprivation-stimulated NHE1 activity and motility (e.g. dn-Rac1, ca-RhoA, dn-RhoA plus deprivation or ca-Rac1 plus deprivation) greatly augmented all of the serum deprivation-induced alterations in cell shape and actin organization. There was an increase in invadopodia in all conditions such as to produce a halo-like effect around the cell. Furthermore, in all conditions that increased NHE1 and motility, except for dn-Rac1, there was increased length and complexity of the pseudopodia that reached its maximum development in the condition of dn-RhoA plus deprivation (see also the larger magnification of the end of an elongated dominant leading-edge pseudopodia; Fig. 6b). A densiometric analysis (AutoDeblur 9.1; AutoQuant Imaging, Inc., New York, NY, USA; data not shown) confirmed that F-actin pixel density decreased with serum deprivation and in those treatments that increased invasion and motility (dn-Rac1, ca-RhoA, dn-RhoA plus deprivation or ca-Rac1 plus deprivation).

### RhoA and Rac1 reciprocally regulate directed motility and invasive capacity of MDA-MB-435 cells via the activity of NHE1

We previously demonstrated that NHE1 activity plays a fundamental role in motility and invasion in MDA-MB-435 cells [6], and so we analyzed the ability of dominant negative and constitutively active mutants of RhoA or Rac1 to modify the serum deprivation-induced motility and invasive ability of MDA-MB-435 cells. As differential apoptosis could affect the outcome of these measurements, the effects of transfection of these mutants of RhoA and Rac1 on apoptosis were analyzed and we observed that neither 1 day of serum deprivation nor transfection of any plasmid had any significant effect on apoptosis in either basal or serum deprived states (data not shown).

**Figure 7:**
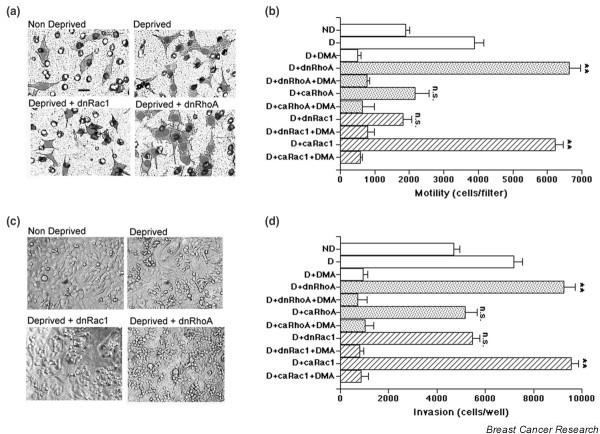
Effect of dominant negative (dn) and constitutively active (ca) RhoA and Rac1 mutants on the Na^+^–H^+ ^exchanger (NHE)1-dependent stimulationof cell motility, invasive capacity and cell shape by serum deprivation. To examine the effect of RhoA and Rac1 on serum deprivation-induced motility and invasive ability of the cells, MDA-MB-435 cell monolayers were left in fresh whole growth medium (nondeprived [ND]) or were serum deprived (D) after having been transfected with dn or ca mutants of RhoA (stippled bars) or Rac1 (stripped bars), or empty vector (empty bars) for 1 day. **(a) **For motility, cells were then trypsinized, centrifuged and resuspended in serum-free medium with or without 2 μmol/l 5-(N, N-dimethyl)-amiloride (DMA). Cells (20,000) were added to the upper chamber of a Boyden Chamber, in which the lower chamber containing medium plus 1% serum was separated by a collagen I coated filter with 8 μm pores. After 4 hours of incubation, filters were removed, processed as described in the Methods section, and the cells that had traversed the filter were photomicrographed (20×). The bar is 10 μm. Circles are the 8 μm pores of of the filter. **(b) **As described in the Methods section, the number of cells that tranversed the collagen I coated filter were counted in photomicrographs taken at 100×. Values are expressed as mean ± standard error. *n *= 4; **P < 0.001 versus nondeprived cells. For invasion, cells were metabolically loaded with ^3^H-thymidine for the 24 hours prior to the experiment, MDA-MB-435 cells were trypinsized, centrifuged and resuspended in serum-free medium, and 30,000 cells were added to the confluent MCF-10A monolayer in each well of a 96 well culture plate. After 8 hours incubation, noninvaded cells were removed and **(c) **the monolayer was photographed at 10× or **(d) **the number of invaded cells determined as described in the Methods section by counting in a Packard TopCount NXT^® ^microplate scintillation counter. Values are expressed as mean ± standard error. *n *= 16; **P < 0.001 versus nondeprived cells.

As shown in Fig. 7, 24 hours of serum deprivation (D) significantly increased basal, non-serum-deprived (ND) MDA-MB-435 cell motility, measured as their ability to cross a collagen I layer in a Boyden Chamber (Fig. 7a,7b) and their capacity to invade a confluent MCF-10A cell monolayer (Fig. 7c,7d). This serum deprivation-dependent stimulation of motility and invasion was strongly inhibited in the cells transfected with either dn-N17Rac1 or ca-V14RhoA and was potentiated in the cells transfected with dn-N19RhoA or ca-V12Rac1. Furthermore, in non-serum-deprived conditions (ND), the basal level of cellular motility was regulated opposite to that observed during deprivation by these mutant constructs. That is, motility was slightly but significantly stimulated by transfection with either dn-N17Rac1 or ca-V14RhoA and slightly but significantly inhibited by transfection with dn-N19RhoA or ca-V12Rac1 (values expressed as cells traversed/filter [mean ± standard error of the mean]: control 2148 ± 80, dn-Rac1 3456 ± 263, ca-RhoA 3055 ± 104, dn-RhoA 1727 ± 94, ca-Rac1 1287 ± 55; *n *= 5 for each treatment).

Altogether, these data strongly suggest that regulation of both motility and invasion closely followed regulation of NHE1 activity. Furthermore, incubation with 2 μmol/l of the specific NHE1 inhibitor DMA almost completely abrogated both basal motility and invasion and their stimulation by serum deprivation, and the potentiation by either dn-N19RhoA or ca-V12Rac1, supporting our previous observation [6] and the findings of others [7-9] that the NHE1 plays a fundamental role in tumour cell motility and invasion. Serum deprived cells that had traversed the collagen coated filter also exhibited a more motile shape than did the serum replete cells (Fig. 7a), and this increase was potentiated in the cells transfected with dn-N19RhoA and attenuated in cells transfected with dn-N17Rac1.

## Discussion

Rho family GTPases are greatly overexpressed in breast tumours [18] and RhoA is necessary for Ras-mediated transformation [19] and metastatic spread [39]. This suggests that these G-proteins play a critical role in neoplastic progression, but the mechanistic basis of their action in tumour cells is still not completely clear [20]. Here we demonstrate that the Rho family of GTPases are involved in the regulation of basal NHE1 activity and especially in the serum deprivation-induced stimulation of its activity. The regulation of both basal NHE1 activity and its upregulation by serum deprivation are linked to a reciprocity of RhoA and Rac1 action (Fig. 4a,4b). We propose that in these breast tumour cells this reciprocal antagonism between RhoA and Rac1 precisely coordinates the specificity and/or magnitude of the pathophysiological response of NHE1 to serum deprivation, with concomitant increases in motility and invasion and hence malignant progression [6].

The reciprocal antagonism between the actions of Rac1 and RhoA on breast cancer cell NHE1 activity, motility and invasive capacity is identical to that reported for movement/migration [21,22] and actin cytoskeleton remodelling [12,40] in other cell systems. Recent evidence supports a prominent role for NHE1 in coordinating motility [6-9] by selectively regulating cytoskeletal events such as focal adhesion assembly and turnover, cortical cytoskeleton dynamics and tyrosine phosphorylation of focal adhesion kinase, with consequent impaired recruitment and assembly of integrins, paxillin and vinculin at focal contacts [41]. Importantly, it was recently demonstrated that NHE1 can regulate these processes through direct interaction with the ERM family cytoskeletal linker protein ezrin [25,26]. In the present study we observed that elevated NHE1 activity is upstream from serum deprivation-dependent actin remodelling (Fig. 2) and the consequent increase in motility and invasion (Fig. 6); furthermore, we found that RhoA and Rac1 act on these processes through a modification of NHE1 activity. This association of increased motility with a decrease in stress fibre number driven by inhibition of RhoA activity is similar to that recently reported for the tight junction protein NZO-3 in controlling renal cell motility [33], and inhibition of motility and invasion by dihydromotuporamine C [34] or by degradation of RhoA by Smurf1 in controlling Mv1Lu cell motility [24], suggesting a common mechanism. In the latter work RhoA degradation was observed primarily in the cell protrusions, and we are currently determining whether inhibition of RhoA by serum deprivation occurs preferentially in the leading edge pseudopodia of the MDA-MB-435 cells.

Altogether these studies strongly suggest that in our cell system the reported antagonistic roles of RhoA and Rac1 in cytoskeletal organization, motility and invasion may also be due to their concerted, convergent action on NHE1 activity. Thus, RhoA and Rac1 integrate cytoskeletal reorganization, which is necessary to direct motility via protein–protein interactions of the NHE1 with other focal adhesion components, and thus they facilitate or inhibit downstream signalling and/or cytoskeletal cascades.

What remains unclear is whether the RhoA-dependent and Rac1-dependent regulation actin organization via NHE1 forms an integral part of the myosin light chain kinase/myosin phosphatase cycle [21,22], or whether it is part of an independent, parallel system that may cooperate to produce the characteristic integration of cell movement by RhoA and Rac1 antagonism. As discussed previously [23], this mechanism concerns smooth muscle cell contraction and cannot fully explain the effects of Rho family proteins on the actin cytoskeleton in other cell types, such as epithelial derived tumour cells. The studies performed to date focused on specific cell types and/or experimental systems, and so it is difficult to determine whether these two mechanisms coexist and to interpret possible interactions between the RhoA/Rac1 modulation of motility via the regulation of NHE1 activity [25,26] or via the myosin phosphatase/myosin light chain kinase cycle [21,22]. Furthermore, it will be necessary to determine whether the redox-dependent downregulation of RhoA by Rac1 with subsequent regulation of cytoskeletion and motility described in HeLa cells [17] has NHE1 as a downstream target. In this way we can begin to acquire an organized view of how the actin cytoskeleton and associated process of motility are regulated by the Rho family of GTPases, and to gain further insights into the mechanisms that underlie the variability of action of these GTPases on actin structures.

## Conclusion

The present study defines the roles of RhoA and Rac1 in the regulation of Na^+^–H^+ ^exchange, and consequently of motility/invasion in tumour cells and in the stimulation of these processes that is unique to tumour cells when confronted with serum deprivation – a common environmental condition in tumours. The regulation of both basal NHE1 activity and its upregulation by serum deprivation are linked to a reciprocity of the actions of RhoA and Rac1. Interestingly, the role of each of these G-proteins is reversed during serum deprivation; basal NHE1 activity is regulated positively by RhoA and negatively by Rac1, whereas RhoA negatively and Rac1 positively directs the stimulation of NHE1 during serum deprivation (Figs 4 and 5).

The findings presented here extend those of our earlier studies documenting the effects of serum deprivation and the role of PI3K on pH regulation, in which we postulated that the observed inversion of PI3K regulatory action contributes, in part, to the upregulation of the NHE1 by serum deprivation in tumour cells [6]. That deprivation-induced switch from positive to negative PI3K regulation may reflect a shift in the coupling of active Rho GTPases to different signalling pathways. We postulate that it is this inversion of RhoA/Rac1 function, together with PI3K regulatory action, that contributes, in part, to the upregulation of NHE1 by serum deprivation in MDA-MB-435 cells, and hence to increased invasion and malignant progression.

To date, the mechanism that drives this shift in RhoA/Rac1 function is not clear but growing evidence indicates that a delicate balance between positive and negative actions of a signal cascade can determine the specificity and/or magnitude of a physiological or pathophysiological response. A particularly important area in biology is the study of mechanisms that underlie dynamic organization of signalling networks in response to specific cellular signals. In the currently developing paradigm, it is becoming ever clearer that specificity of response in signal transduction is attained through the compartmentalization of signalling proteins and effectors close to their activators, regulatory elements and targets to ensure tight regulation and specificity of action of signalling cascades [39]. The assembly of these multimolecular complexes or modules at the cellular site of action is accomplished by scaffolding proteins that have the capacity, through simultaneous interaction with multiple signalling proteins, to integrate diverse signalling pathways [40]. A possibility is that serum deprivation alters the balance of expression or activity of a scaffolding protein, resulting in the creation of new spatiotemporal combinations and/or altered integration of signal components. Finally, the reversal in the roles of Rac1 and RhoA in the regulation of NHE1 activity and motility observed here might explain some seemingly contradictory reports in which activating RhoA can lead to either increased or decreased motility and invasiveness [18]. These discrepancies possibly reflect different serum treatments of the tumour cells after RhoA activation and point out the peril of an across-the-board inhibition of RhoA as a possible therapy. Importantly, these results also unify recent advances concerning the mechanisms underlying tumour invasive potential: the RhoA-ROCK activation of NHE1 [41,42], the RhoA-ROCK mediation of invasion [43,44] and role of the NHE1 in driving tumour cell motility [6-9] and invasion [6]. Further studies are now needed to elucidate the signal transduction components that lie upstream and downstream of RhoA and Rac1 action, and to clarify the interactions between the RhoA-and Rac1-dependent signal transduction pathways that are involved in their reciprocal antagonism. It will be of key interest to investigate the interrelationships between these activities and to define the effectors and downstream mediators that are involved in RhoA-and Rac1-transduced mitogenesis, transformation and invasiveness.

## Competing Interests

None declared.

## Abbreviations

ca = constitutively active; dn = dominant negative; DMA = 5-(N, N-dimethyl)-amiloride; DMEM = Dulbecco's modified Eagle's medium; GST = glutathione S-transferase; HA = haemagglutinin; NHE = Na^+^/H^+ ^exchanger; PAK = p21-activated kinase; PBS = phosphate-buffered saline; pHi = intracellular pH; PI3K = phosphoinositide-3 kinase; RBD = Rho-binding domain of Rhotekin; ROCK = Rho kinase.

## Figures and Tables

**Table 1:** Effect of serum deprivation on the kinetic parameters of the allosteric proton regulatory site in human breast cell monolayers

Kinetic parameter	ND	D	D + dn-RhoA	D + dn-Rac1
Vmax	19.6 ± 0.75	26.2 ± 0.86*	31.3 ± 0.88^†^	21.6 ± 0.9
K' for H^+^	0.150 ± 0.02	0.113 ± 0.05^†^	0.105 ± 0.07^†^	0.137 ± 0.023
n_app_	2.19	2.16	2.03	2.23

Values are expressed as mean ± standard error for Vmax (mmol/l H^+^/minute) and H^+ ^apparent K' (μmol/l) measured at 135 mmol/l external sodium. The Hill coefficient (n_app_) was derived at 135 mmol/l external sodium. Confluent cell monolayers were either kept in serum complete medium (nondeprived [ND]) or were placed in serum free growth medium (D) minus or plus dominant negative (dn)-RhoA or dn-Rac1 for 24 hours prior to measurement of ΔH^+^. *n *= 5; *P < 0.01, ^†^*P *< 0.001, versus ND.
